# Overview of BITS2005, the Second Annual Meeting of the Italian Bioinformatics Society

**DOI:** 10.1186/1471-2105-6-S4-S1

**Published:** 2005-12-01

**Authors:** Manuela Helmer-Citterich, Rita Casadio, Alessandro Guffanti, Giancarlo Mauri, Luciano Milanesi, Graziano Pesole, Giorgio Valle, Cecilia Saccone

**Affiliations:** 1 Centre for Molecular Bioinformatics, Dept of Biology, University of Rome Tor Vergata, Rome, Italy; 2 Department of Biology, University of Bologna, Italy; 3 FIRC Institute of Molecular Oncology, Milan, Italy; 4 Department of Computer Science, Systems and Communication, University of Milan-Biococca, Milan, Italy; 5 Institute for Biomedical Technologies, National Research Council (ITB-CNR), Milan, Italy; 6 Dept of Biomolecular Sciences and Biotechnologies, University of Milan, Milan, Italy; 7 Department of Biology and CRIBI Biotechnology Centre, University of Padua, Padua, Italy; 8 University of Bari and ITB - CNR, Bari, Italy

## Abstract

The BITS2005 Conference brought together about 200 Italian scientists working in the field of Bioinformatics, students in Biology, Computer Science and Bioinformatics on March 17–19 2005, in Milan. This Editorial provides a brief overview of the Conference topics and introduces the peer-reviewed manuscripts accepted for publication in this Supplement.

## Introduction

The ITalian Bioinformatics Society (B.IT.S.; ) aims at bringing together research scientists interested in Bioinformatics, as a multi-disciplinary science for studying biological systems at the molecular and cellular level by using informatics and computational methods and models. The main goals of B.IT.S. are the study, development and spread of Bioinformatics throughout the Italian scientific, academic, technological and industrial community.

The Society was founded in 2003 after 5 years of enjoyable and fruitful informal meetings. It attracts the interest and efforts of scientists from different research areas in Italy: Molecular Biology, Biochemistry, Physics and Computer Science.

The Second Annual BITS Meeting  was held at the Leonardo Da Vinci Hotel Congress Center in Milan on March 17–19th, 2005. David Lipman, Director of NCBI, Shoshana Wodak, from the Department of Biochemistry, University of Toronto, Canada, and Mikhail Gelfand, from the Institute for Information Transmission Problems, RAS, Russia were the keynote speakers at the BITS2005 Conference. Dr. Lipman opened the meeting with the "Giuliano Preparata Lecture", dedicated to a brilliant and innovative Italian physicist who also made important contributions in the field of Computational Biology, and, unfortunately, passed away a few years ago. Dr. Lipman's talk was about the Semantic Shift in Comparative Genomics & Systems Biology and started an interesting discussion about the new developments and challenges that Bioinformatics will have to face in the near future. Mikhail Gelfand talked about the evolution of riboswitches, while Shoshana Wodak gave a talk entitled Protein-Protein Interactions: The challenge of prediction specificity, and spoke from a historical perspective with interesting comments about the latest developments in the field.

The conference was organized into thematic sessions: Comparative Genomics, Database and Data Mining, Structural Bioinformatics, Algorithms and Applications, Functional Bioinformatics, Medical Bioinformatics. Abstracts were collected on the different topics and selected for oral presentations; some of them were then submitted as complete research papers for this Supplement. The manuscripts were edited by a committee composed of the Meeting Organizers and the Steering Committee of the Italian Bioinformatics Society and then sent for peer review to a panel of non-Italian referees.

The manuscripts cover several topics central to the development of Computational Biology, such as the development and usage of resources for analysing expression data, tools for the analysis of protein structure, interaction and networks, and the development of informatics resources for biological data integration.

## Genome analysis

L. Milanesi *et al*. [1] describe a systematic analysis of the human genome in the search for proteins containing kinase catalytic domains. The predicted set of human kinases, the human *kinome*, was extended by identifying both additional genes and potential splice variants. The results of the research are collected in the KinWeb database, available at the address .

The ESTree database by Lazzari *et al*. [2], collecting both in house prepared cDNA libraries and publicly available *Prunus persica *expressed sequence tags (ESTs), represents a useful resource for peach functional genomics. The database contains more than 18,000 sequences, with links to predicted SNPs, GO terms, the NiceZyme and KEGG pathway databases.

## Sequence analysis

A number of papers describe tools designed for sequence analysis.

ParPEST (Parallel Processing of ESTs) is a pipeline based on parallel computing for EST analysis [3]. It relies on distributed processes and parallelized software.

The GFINDer resource [4] was presented with new modules for performing phenotype analyses of inherited disorder related genes [5]. New GFINDer modules make it possible to annotate large numbers of user classified sequence identifiers with morbidity and clinical information, classifying them according to genetic disease phenotypes and their locations of occurrence, and statistically analyzing the obtained classifications.

Fariselli *et al*. [6] describe the implementation of the posterior-Viterbi (PV) algorithm for the prediction of the topology of all-beta membrane proteins. They show that PV decoding performs better than other algorithms when tested on the problem of predicting the topology of beta-barrel membrane proteins.

A neural network approach is described in Ferraro *et al*. [7] for the inference of SH3 domain specificity. The network performs better than regular expressions and PSSMs in the detection of SH3 domains interactors. The authors show that this approach, however, is dependent upon the number of binding peptides used in the training set.

## Phylogenetics

*Caenorhabditis elegans *and *C. briggsae *share conserved but rapidly evolving genomes. Rambaldi *et al*. [8] developed NemaFootPrinter (Nematode Transcription Factor Scan Through Phylogenetic Footprinting), a web-based software for interactive identification of conserved, non-exonic DNA segments in their genomes. This software relies on the identification of orthologous genes and on the manual selection of gene boundaries. The resource is available at the address: .

## Structural bioinformatics

Secondary structure prediction is considered an interesting topic *per se *and also as an ancillary method to structure prediction methods. Armano *et al*. [9] describe a hybrid technique for secondary structure prediction, based on a "sequence-to-structure" prediction, enforced by resorting to a population of hybrid (genetic-neural) experts, and then on a "structure-to-structure" prediction, by means of an artificial neural network. This new technique attains 76% accuracy, comparable with other state-of-the-art methods.

Secondary structure assignment also represents a fundamental issue, both for classification purposes and for improving the potential of prediction methods. Cubellis *et al*. [10] developed an accurate method for assigning secondary structure based on main chain geometry. The SEGNO program is compared with other pre-existing methods. It defines more types of secondary structure (i.e. poly-proline and 3–10 helices). Moreover, amino-acid trends at helix caps are stronger, secondary structural elements are less likely to be concatenated together, and secondary structure guided sequence alignment is improved.

Ausiello *et al*. [11] describe the Query 3D program for local comparison of protein structures. Query 3D is at the core of the pdbFun server [12] for the identification of local structural similarities between annotated residues in proteins.

D'Ursi *et al*. [13] used a flexible docking approach to characterize the molecular interaction between seven endocrine disrupting chemicals and estrogen, progesterone and androgen receptors in the ligand-binding domain. All ligands docked in the buried hydrophobic cavity corresponding to the hormone steroid pocket. The results are in agreement with known toxicological data and suggest a hydrophobic cavity is needed to accommodate the analyzed chlorine-carrying compounds.

In [14], Greco *et al*. analyze the relatively rare double histone fold, which is tightly related to the structure of nucleosomal histones. Through the application of several secondary structure prediction and fold recognition methods, they showed that the viral protein gi|22788712 is compatible with the structure of a H3-H4-like histone pseudodimer and may retain the ability of mediating protein-DNA interactions.

## Gene expression

Several papers in this Supplement concentrate on functional genomics and the analysis of expression data, proving the field's vitality and the experimental laboratory's interest in involving the bioinformatic community in their research.

Ancona *et al*. [15] compare the Regularized Least Squares (RLS) and Support Vector Machines (SVM) approaches in cancer classification through the analysis of microarray data. They show that the results of the two methods are comparable, even if RLS may represent a valuable alternative due to their simplicity and low computational complexity.

Burgarella *et al*. [16] developed MicroGen, a web system for managing information and workflow in the production pipeline of spotted microarray experiments. MicroGen is composed of a core multi-database system able to store all data from different spotted microarray experiments according to the Minimum Information About Microarray Experiments (MIAME) standard. It offers an intuitive and user-friendly web interface able to support collaborative work among the multidisciplinary actors and roles involved in spotted microarray experiment production.

The analysis of two transcription profiles led Cavallo *et al*. [17] to the identification of five Tumor Associated Antigens (TAA), whose expression is linearly related to the tumor mass increase in BALB-neuT mammary glands. Normal expression of these proteins is low and compatible with the design of immunopreventive vaccines.

Finocchiaro *et al*. [18] describe an interesting approach, based on data extracted from biomedical literature and the analysis of Gene Ontology categories. Such data can be used to complement expression data in order to highlight microarray datasets biologically related in gene expression.

Di Camillo *et al*. [19] describe and test a quantization method, based on a model of the experimental error and on a significance level able to mediate between false positive and false negative classifications in the analysis of microarray data. The quantization method, evaluated in comparison with the two standard methods, improves the ability of Reveal and Dynamic Bayesian Networks to identify relations between genes.

## Genetic and population analysis

The Hmt database is presented by Attimonelli *et al*. [20], as a well-integrated web-based human mitochondrial bioinformatic resource aimed at supporting population genetics and mitochondrial disease studies. HmtDB consists of a database of Human Mitochondrial Genomes, annotated with population data, and a set of bioinformatic tools. It is able to produce site-specific variability data and to automatically characterize newly-sequenced human mitochondrial genomes.

The PedNavigator tool [21], specifically designed by Mancosu and co-workers for genetic studies, is a browser for genealogical databases. It is useful for genealogical research due to its capacity to represent family relations between individuals and to make a visual verification of the links during family history reconstruction. As for genetic studies, it is helpful to follow propagation of a specific set of genetic markers (haplotype), or to select people for linkage analysis, showing relations between various branches of an affected subjects' family tree.

## Systems biology

High-throughput approaches have been applied to the analysis of protein interaction in several model organisms, but have not yet been attempted in humans, where the unraveling of the interactome is one of the most ambitious tasks facing proteomics. An inferred human protein interaction network was built by Persico *et al*. [22], based on the identification of reliable orthologues of proteins known to interact in a number of reference sets. The HomoMINT resulting network is stored in the MINT database [23].

## Data and text mining

A high performance workflow is described by Merelli *et al*. [24]. Using grid technology, it correlates different kinds of bioinformatic data, from the nucleotide sequence to the exposed residues of the protein surface. The proposed workflow is implemented to integrate huge amounts of data. The results must be stored in a relational database.

## Databases and ontologies

Romano *et al*. [25] describe a system that is able to access and execute predefined workflows. Web Services allow access to the IARC TP53 Mutation Database [26] (containing all TP53 gene mutations identified in human cancers and cell lines that have been reported in the peer-reviewed literature since 1989) and CABRI (Common Access to Biological Resources and Information) catalogues of biological resources.

An SRS site, with both EMBL and CABRI catalogues, has been set up by Romano *et al*. [27]. In the site about 67,500 valid cross-references were identified between the two databases. Such links were added to the EMBL Data Library and now make it possible to establish further links between the CABRI catalogues and other bioinformatic databases cross-referenced in the EMBL database.

## Future meeting

The next Annual meeting of the Italian Bioinformatics Society will be held in Bologna in Spring 2006. Further information about BITS2006 will be available on our web site at the address .
